# Vesicoureteral Reflux, Reflux Nephropathy, and End-Stage Renal Disease

**DOI:** 10.1155/2008/508949

**Published:** 2008-07-21

**Authors:** Paul Brakeman

**Affiliations:** Division of Pediatric Nephrology, Department of Pediatrics, University of California, San Francisco, CA 94143-0532, USA

## Abstract

*Objective*. To review the contribution of vesicoureteral reflux and reflux nephropathy to end-stage renal disease. *Data Source*. Published research articles and publicly available registries. *Results*. Vesicoureteral reflux (VUR) is commonly identified in pediatric patients and can be associated with reflux nephropathy (RN), chronic kidney disease (CKD), and rarely end-stage renal disease (ESRD). Patients with reduced GFR, bilateral disease, grade V VUR, proteinuria, and hypertension are more likely to progress to CKD and ESRD. Because progression to ESRD is rare in VUR and often requires many decades to develop, there are limited prospective, randomized, controlled trials available to direct therapy to prevent progression to ESRD. *Conclusions*. Identification of patients with increased risk of progression to CKD and ESRD should be the goal of clinical, biochemical, and radiological evaluation of patients with VUR. Treatment of patients with VUR should be directed at preventing new renal injury and preserving renal function.

## 1. INTRODUCTION

Vesicoureteral reflux (VUR) is a common finding in pediatric patients. Approximately 1/3 of patients who have had a urinary tract infection (UTI) have VUR and 9–20% of patients with prenatal hydronephrosis have VUR when tested postnatally [1]. The prevalence of VUR in the general pediatric population has been estimated recently to be as high as 17.2% [1, 2]. Some patients with VUR develop reflux nephropathy (RN), some patients with RN develop chronic kidney disease (CKD), and a small number of patients progress to end-stage renal disease (ESRD). While UTI and VUR are relatively common, ESRD is rare in the pediatric population with an unadjusted incident rate of 14.8 per million patients per year in 2005 for ages 0–18 years [3]. The goal of this article is to describe the contribution of VUR to ESRD in pediatric patients, define risks for progression, and review data indicating what treatments may prevent progression to ESRD for patients with VUR.

## 2. RENAL PATHOPHYSIOLOGY IN REFLUX NEPHROPATHY

The mechanisms for the development of ESRD in VUR are complex. In animals, when the flow of urine is obstructed in the developing kidney a series of abnormalities occur including (1) arrest of glomerular maturation, (2) glomerulosclerosis, (3) ischemia and necrosis of some tubular cells, (4) apoptosis of other tubular and collecting duct cells, (5) interstitial inflammation, proliferation, and fibrosis, and (6) tubular dilatation and atrophy [4–6]. In addition, in animals and humans there is evidence that scarring occurs in compound papillae where intrarenal reflux is present [7]. In humans, RN is usually identified as renal scarring as defined on dimercaptosuccinic acid (DMSA) scan in a patient known to have VUR. It is important to note that the causality is not completely clear as some patients have renal scarring by DMSA scan but do not have VUR. It is also clear that pyelonephritis in the presence of VUR may lead to new scarring on DMSA scans; however, some patients with VUR have RN with renal scarring by DMSA scan at the time of diagnosis whether or not they have had a urinary tract infection. This is highlighted by the fact that some patients diagnosed at birth have renal scarring as defined by DMSA scan [8, 9]. One possible explanation for this is that damage to the kidney may occur embryonically due to VUR. Alternatively, some of the genes that control normal development of the ureters and ureterovesicular junction also control renal development. Thus VUR may be associated with either macroscopically abnormal renal development or subtle developmental changes that predispose the kidney to developing scarring as identified by DMSA scan. A portion of the patients who develop ESRD related to RN may have abnormally developed kidneys that progressively worsen over time with further decrease in renal function exacerbated by proteinuria, hypertension, and episodes of pyelonephritis. This is highlighted by the fact that in multiple studies, correction of VUR does not completely prevent the formation of new scars [10, 11], indicating that there may be worsening renal pathology even once VUR has been corrected in some patients.

## 3. REFLUX NEPHROPATHY IS A MAJOR CAUSE OF ESRD IN CHILDREN

Multiple registries in the United States and internationally have identified RN as an important cause of ESRD. For adults, RN is not a very common cause of ESRD in children. In the USRDS database, RN is not specifically listed as an etiology for ESRD; however, obstructive uropathy not due to ureteropelvic junction or ureterovesicular junction obstruction is one of the less common causes for ESRD. For all ages, obstructive uropathy accounted for 0.6% of the point prevalent cases for 2005 [3]; whereas diabetes accounted for 36%. The incidence of obstructive uropathy in the USRDS has been stable at approximately 0.3% since 1994 [12], but has increased from 0.1% for all ages for 1989–93 [13] to 0.3% for the time periods 1994–98 [12], and 1999–2003 [3]. In the north American pediatric population, RN is reported as the 4th leading cause for dialysis and transplantation with 5.3% of transplant patients having a diagnosis of RN and 3.5% of dialysis patients having a diagnosis of RN [14]. The incidence of RN in the pediatric population has remained stable from 2003 to 2007 [14, 15]. It is important to note that the 2nd and 3rd leading causes for dialysis and transplantation in children are obstructive uropathy and aplasia/hypoplasia/dysplasia either of which can be intertwined with RN [14]. Furthermore, in this pediatric population another 2.6% of the transplant patients and 2% of dialysis patients carry a diagnosis of prune belly syndrome which is a disease of urinary obstruction in uteroand is often associated with VUR [14]. The accuracy of these registries is dependent on those entering data and diagnostic codes and thus may overrepresent or underrepresent the importance of RN in ESRD. However, in various international reports reflux nephropathy either alone or in combination with congenital obstructive disease also is identified consistently as a leading cause of ESRD [16–21].

## 4. VUR IS COMMON IN CHILDREN; HOWEVER, ESRD RELATED TO VUR IS RARE

In the North American Pediatric Renal Trials and Collaborative Studies registry, RN accounts for approximately 5% of the pediatric ESRD population [14]. It is possible to dispute the accuracy of this figure as this registry depends on voluntary reporting of data and there is no verification of the accuracy of the assigned diagnoses. However, if one uses this figure as an estimate and combines it with the annual incidence of ESRD for ages 0–18 reported by the USRDS of 14.8 per million, then the incidence of ESRD related to RN in the pediatric population would be approximately 0.7 per million patients [3, 14]. If one compares this annual incidence to the estimated prevalence of VUR in the general population, which has been recently reported as 17.2% or 172 000 per million, it is clear that the vast majority of patients with VUR do not develop ESRD. Even if one uses older estimates of the prevalence of VUR in the general population of 1-2% or 10 000 to 20 000 per million patients [2, 22], VUR is much more common than ESRD. Since the most common type of VUR is low-grade VUR or grades I–III VUR, this implies that lower grade reflux very rarely is associated with decreased renal function. Given that most patients with VUR do not develop ESRD or even CKD, much work has centered on identifying those patients with VUR who are at risk of developing CKD and ESRD. This work has been complicated by the fact that many older reports on outcomes of VUR were based on datasets from referral centers, not the general pediatric population, and thus are likely to have a strong bias towards patients with more severe disease.

## 5. RISK FACTORS FOR PROGRESSION TO CKD AND ESRD IN PEDIATRIC PATIENTS WITH VUR

Multiple retrospective trials have identified factors predictive of progression to CKD and ESRD in pediatric patients with VUR (Table 1).

There have been few papers that have focused solely on progression to ESRD as a primary endpoint in patients with RN, since, as described above, ESRD in general is a rare event for patients with VUR. Table 1 lists studies describing risk factors for CKD and ESRD in patients with VUR. Ardissino et al. retrospectively evaluated the risk of progressing from CKD to ESRD in a cohort of 322 pediatric patients with VUR and creatinine clearance (CrCl) <1.25 mL/s per 1.73 m^2^ body surface area and found an overall risk of 56% for progressing to ESRD by the age of 20 [21]. Not surprisingly, those patients with CrCl <0.67 mL/s per 1.73 m^2^ had a 4-fold increased risk of progressing to ESRD compared to those with CrCl ≥ to 0.67 mL/s per 1.73 m^2^. In addition, age at diagnosis was not associated with an increased risk of progression to ESRD with those diagnosed at age greater than 6 months having no significant difference in risk of progression to ESRD compared to those diagnosed at age ≤6 months. In this cohort, grade IV reflux was the most common grade of VUR; however, information on the grade of VUR was reported for only 51% of the patients, making it difficult to relate risk of progression to grade of reflux. 29.1% of the patients were either hypertensive or being treated with antihypertensive medication, demonstrating the association between hypertension and RN. In addition, 104 of the 322 patients were evaluated for proteinuria, and approximately 1/3 (34/104) had moderate to severe proteinuria (uPr/uCr 0.95–7.2). Those patients with moderate to severe proteinuria showed a statistically significant larger mean rate of CrCl decrease when compared to those with mild or no proteinuria, thus demonstrating that proteinuria is associated with ongoing renal deterioration and may be a target for therapies to prevent progression to ESRD.

Because having CKD increases the risk of progressing to ESRD in patients with VUR, risk factors for progressing to CKD are highly likely to be significant predictors for the progression to ESRD. Several studies have focused on risk factors for developing CKD in patients with VUR. Silva published data on a retrospective cohort of 735 pediatric patients with VUR of all grades with 29% of the patients having high-grade VUR (grades IV and V) [27]. Thus, this cohort exhibited some selection bias as the rate of high-grade VUR was significantly higher than reported in studies in the general population. In this cohort, 3% developed CKD (as defined by GFR <1.25 mL/s per 1.73 m^2^ body surface area as estimated by the Schwarz formula) and 1.5% developed ESRD (GFR <0.25 mL/s per 1.73 m^2^). Progression to CKD was strongly associated with hypertension. As part of the same work, Silva et al. evaluated 184 pediatric patients with severe bilateral reflux (grades III–V) followed at a single tertiary care center [28]. Mean follow-up was 78.6 months. All patients received daily antibiotic prophylaxis and 15% (27/184) had surgical reimplantation. In this higher-risk cohort, the estimated probability of developing CKD was approximately 15% at 10 years postdiagnosis of VUR. In multivariate analysis, age at diagnosis >24 months, VUR grade V, and bilateral renal damage were associated with an increased risk for CKD. Interestingly, diagnosis of VUR after 1990 was associated with reduced risk for CKD. This data implies that our current diagnosis and treatment of VUR may reduce the risk of developing CKD. In addition, the estimated risk of CKD was 0% for patients with grade III reflux or a negative DMSA at the time of diagnosis. The lack of progression to CKD in those patients with a normal DMSA at diagnosis implies that, perhaps, it is only those kidneys with congenital lesions or that already have been significantly damaged at diagnosis that are at risk for development of significant renal impairment.

Several other studies also have focused on high-risk populations of VUR patients. Neild et al. evaluated a high-risk population of 44 patients with bilaterally scarred kidneys due to primary reflux or bladder dysfunction and GFR 0.25–1.0 mL/s per 1.73 m^2^ based on either an eGFR using the Jelliffe formulae or plasma clearance of EDTA [24]. They identified a watershed GFR of 0.83 mL/s per 1.73 m^2^ below which the likelihood of progressing to ESRD increased substantially. In addition, they identified proteinuria as predictive of increased risk of CKD and were able to demonstrate a protective effect of ACE inhibitors on the rate of decline of renal function for patients with eGFR > 0.75–0.83 mL/s per 1.73 m^2^. Caione et al. retrospectively reviewed 50 patients from Italy with bilateral VUR grades III-V diagnosed in the first year of life with an average follow-up of 6.3 years [23]. In their cohort, 54% of the patients developed CKD as defined by eGFR <1.3 mL/s per 1.73 m^2^. All were boys, and in multivariate analysis neither number of UTIs nor prenatal diagnosis modified the likelihood of CKD. In multivariate analysis, a serum creatinine >53 umol/L significantly increased the likelihood of developing CKD. These two studies, while both small, appear to demonstrate that there is a threshold after which renal function declines with much greater frequency to CKD. In addition, Caione's study did not identify an association of CKD with febrile UTIs, implying once again that, perhaps, patients with severe VUR progress to CKD due to ongoing inflammation and pathologic changes or developmental abnormalities rather than acquired damage.

## 6. VERY LONG-TERM FOLLOW-UP OF VUR PATIENTS

There have been several other retrospective cohort studies from a variety of populations with very long follow-up that evaluated the long-term outcome of VUR. For these patients with very long follow-up, treatment was initiated in some as long as 40 years ago, and it is possible that current treatment protocols, including more aggressive treatment of voiding dysfunction, may yield different outcomes than treatment practices from 40 years ago. In addition, these cohorts from several decades ago also appear to share a selection bias towards patients with more severe VUR and higher rates of scarring, perhaps because only the most severe VUR with recurrent infections was diagnosed in the past. Also, renal scarring was identified by intravenous pyelogram which is not as sensitive as DMSA scans; thus, patients identified as having renal scarring had more severe renal damage. Lahdes-Vasama et al. evaluated a cohort of Finnish patients followed for an average of 37 years [25]. They attempted to enroll 267 patients with VUR diagnosed between 1955 and 1965 but only were able to report information on current renal function for 127 of the patients. In this cohort, 12/265 had died due to kidney-related conditions, 7/265 had undergone renal transplantation, and 1/265 was on hemodialysis. For those who agreed to enroll, 85/127 had GFR <1.5 mL/s per 1.73 m^2^, 4/127 had GFR <60 mL/min/1.73 m^2^, and 1/127 had GFR <0.50 mL/s per 1.73 m^2^ based on the Cockcroft-Gault formula. Among the enrolled patients, 35% had unilateral scarring and 24% had bilateral scarring by ultrasound, and the patients with bilateral scarring were significantly more likely to have reduced GFR. Interestingly, this Finnish cohort had no increased prevalence of hypertension compared to the rest of the Finnish population. The study implies that approximately 7% of patients with VUR progress to ESRD; however, the study was limited because they were unable to evaluate grades of reflux or the presence or treatment of voiding dysfunction, and there was a very high rate of renal scarring that was severe enough to be measurable by ultrasound. These factors indicate that there may have been significant selection bias in this cohort.

El-Khatib et al. reported data from 293 patients who were diagnosed with RN or VUR between 1971 and 1986 in Australia [29]. In this group, most patients were females who presented with febrile UTI; there was no information on VUR grade; and 89% of the patients had renal scarring on IVP. Thus, this population was highly selected for patients with more severe RN than a general population of patients with VUR. In this cohort, 37% demonstrated deterioration in renal function based on rising serum creatinine. In multiple regression analysis, the independent risk factors for rise in serum creatinine were proteinuria, hypertension, elevated creatinine at presentation, bilateral VUR, and male sex. Zhang and Bailey presented retrospective data on 294 (59 males) patients over 15 years of age who had been followed on average for more than 10 years. At last follow-up, 24% had creatinine clearance <1.2 mL/s per 1.73 m^2^ [30].

There have been several other smaller long-term follow-up studies published. Mor et al. reported data from 100 Israelis (79 women and 21 men) followed for more than 20 years post antireflux surgery [26]. In their cohort, only 1/100 patients had an abnormal serum creatinine level; however, eGFR was not reported, no information on voiding dysfunction was reported and there was no information on VUR grade. Given these limitations, this study indicates a low risk of progression to ESRD for their cohort. Arze et al. presented data from 130 patients (16 male) identified in 1976 as having renal scarring as defined by IVP or pathologic evaluation of renal tissue post nephrectomy [31]. In their cohort which was followed for up to 240 months, 18% had, or developed, CKD as defined by Cr >130 uM/L. Hypertension, proteinuria, and repeated UTI were associated with increased GFR. Nakashima et al. followed 95 patients who had renal scar or grade III or higher VUR and found that 3/995 developed ESRD and that 35% demonstrated renal function deterioration [32]. In their cohort, bilateral scarring, proteinuria >300 mg per day, diastolic hypertension, and low GFR (mean 0.82 mL/s per 1.73 m^2^) were associated with increased risk of deterioration of renal function.

## 7. PREVENTION OF ESRD IN PATIENTS WITH VUR

**Figure 1:**
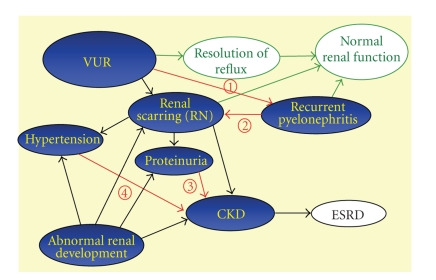
Schematic representation of factors involved in progression to ESRD for patients with VUR. In the majority of patients with VUR, VUR resolves and the patients demonstrate normal renal function (green pathway). Some patients with renal scarring and/or who have recurrent pyelonephritis also retain normal renal function (green arrows). Other patients with VUR develop RN, proteinuria, and hypertension. In all cases, abnormal renal development can accompany RN and contribute to renal scarring, proteinuria, hypertension, and progression to CKD (solid black arrows). Prevention of ESRD focuses on intervening to prevent recurrent pyelonephritis (1), by actively evaluating and treating episodes of pyelonephritis to prevent renal scarring (2), and by treating hypertension (3) and proteinuria (4) to preserve renal function.

Currently, there is little evidence from prospective, randomized controlled trials to direct therapies to prevent ESRD in patients with VUR. One goal of treatment is to try to prevent recurrent episodes of pyelonephritis and renal scarring by treating voiding dysfunction, surgically correcting VUR, using daily antibiotic prophylaxis and treating episodes of pyelonephritis quickly and effectively [33] (see Figure 1). All patients should be completely evaluated and treated for voiding dysfunction as part of the evaluation and treatment of VUR in order to maximize bladder function and preserve renal function. Randomized controlled trials that have tested the benefit of surgical correction of VUR or prophylactic antibiotic treatment have not demonstrated either is more efficacious in preventing renal scarring or the overall rate of recurrent UTIs [10, 11]. Critically, these studies did not have a control group that received only observation. In one of these trials, the International Reflux Study in Children trial, surgical correction of grades III and IV reflux did reduce the occurrence of febrile UTI. Unfortunately, this did not correspond to a decrease in new renal scars or an improvement in renal function in surgically treated children [10]. Several recent reports have questioned the utility of daily antibiotic prophylaxis [34–36]; however, it is important to note that the studies from Garin et al. [35] and [34] Conway et al. reported on few male subjects and did not address high-grade VUR. Specifically, the Garin trial excluded those with VUR grades IV and V, and the Conway study included only 10 patients with VUR grades IV and V. Another recent randomized prospective trial demonstrated a benefit of prophylactic antibiotics versus observation in preventing positive surveillance urine cultures in asymptomatic boys with grade III VUR [37]. In the near future, we will hopefully have new data from a large, multicenter trial comparing daily antibiotic prophylaxis versus observation in patients with low-grade reflux [38]. At this point in time, there does not appear to be good evidence to support using daily antibiotic prophylaxis to prevent UTI or renal scarring in patients with VUR grades I–III; nor is there evidence that for grades III-IV VUR surgical correction of VUR prevents new renal scarring compared to daily antibiotic prophylaxis. Because grade V VUR is rare, there have not been any significant randomized, controlled, prospective trials to evaluate treatment options. Thus, the treatments that may prevent ESRD in this high-risk population are incompletely characterized. For patients with high risk of progression to CKD and ESRD such as those with grade IV and V reflux, significant renal scarring and those with reduced GFR, the surgical correction of reflux and daily antibiotic prophylaxis should be strongly considered; and risks and benefits of these treatments should be discussed with families. In addition, close clinical follow-up and rapid treatment of episodes of pyelonephritis should be instituted to preserve renal function and prevent progression to ESRD.

## 8. HYPERTENSION AND PROTEINURIA AS THERAPEUTIC TARGETS FOR PREVENTION OF ESRD

Another aspect of preventing the progression of RN to ESRD is the treatment of hypertension and proteinuria, both of which are indicators of renal damage and contribute to ongoing deterioration of renal function in many renal conditions. As described above, multiple studies have demonstrated a correlation between RN and hypertension. Hypertension has been shown to affect the rate of decline of renal function in other conditions, thus controlling hypertension should be a significant goal for treatment of patients with VUR.

In addition, multiple studies have demonstrated a correlation between proteinuria and risk for CKD in RN. The magnitude of proteinuria associated with increased risk of CKD or deterioration of function varies somewhat but even mild proteinuria appears to be associated with increased risk for renal deterioration. El-Khatib et al. showed an increased risk of deterioration of renal function for patients with >0.2 G per day of proteinuria with a progressively increasing risk of deterioration for patients with >1 G per day of proteinuria [29]. Nakashima et al. demonstrated an increased risk for deterioration of renal function for patients with >0.3 G/day of proteinuria [32]. Neild et al. also demonstrated a correlation between increased proteinuria and elevated creatinine with patients having a GFR of 0.25 mL/s per 1.73 m^2^ to 0.5 mL/s per 1.73 m^2^ having an average protein to creatinine ratio of 209 mg/mmol compared to 38 mg/mmol for those patients with GFR of 0.83 mL/s per 1.73 m^2^ to 1.0 mL/s per 1.73 m^2^[24].

Neild et al. also presented the only data in VUR patients that ACE inhibitors may be able to slow the progression of renal deterioration associated with severe RN [24]. One caveat to their finding was that benefit of ACE inhibition was demonstrated only for those patients with mildly reduced GFR of 0.83 mL/s per 1.73 m^2^ to 1.0 mL/s per 1.73 m^2^ [24]. There is evidence that in nondiabetic patients with renal parenchymal abnormalities that ACE inhibition reduces proteinuria and may help to preserve renal function [39–41]. Given the benefit of ACE inhibition in other renal conditions and the limited, but promising, data presented by Neild et al. [24], ACE inhibitors and/or angiotensin receptor blocking agents should be the first choice for controlling hypertension and proteinuria and should be initiated early in the course of disease. Furthermore, based on data from other nondiabetic renal disease, one should use ACE inhibition and/or angiotensin receptor blockade even in the absence of hypertension when a patient has VUR and proteinuria. Controlling hypertension and proteinuria in patients with VUR should be considered standard maintenance therapy for those with VUR and RN.

## 9. CONCLUSIONS

VUR is commonly identified in pediatric patients and can be associated with reflux nephropathy, CKD, and, rarely, ESRD. The progression of RN to CKD and ESRD is more likely in patients with reduced GFR, bilateral VUR and/or renal scarring, grade V VUR, proteinuria, and hypertension. Identification of patients with these clinical characteristics should be the goal of clinical, biochemical, and radiological to evaluation of patients presenting with hydronephrosis on prenatal ultrasound or febrile UTI. Because progression to ESRD is rare in VUR and often requires many decades to develop, there are limited prospective, randomized, controlled trials available to direct therapy. All patients should be evaluated and treated for voiding dysfunction, where appropriate, and rapidly diagnosed and treated for recurrent pyelonephritis. Evaluation and treatment of patients with VUR should be directed at preventing pyelonephritis and new renal injury; however, there is little evidence that either surgical correction of VUR or antibiotic prophylaxis prevents pyelonephritis and new renal scarring in comparison to careful clinical observation alone. In addition, for those patients who do develop RN, care should be taken to normalize blood pressure and reduce proteinuria in order to preserve renal function. In the future, with continued basic research, we may be able to develop pharmaceutical therapies aimed directly at the molecular pathophysiology of RN to slow progression of RN to ESRD. For now, we must provide the best supportive care to patients to preserve renal function and prevent ESRD in patients with vesicoureteral reflux and reflux nephropathy.

## Figures and Tables

**Table 1:** Characteristics of studies reporting CKD and ESRD data for VUR.

Study	N (males)	Mean length of F/U in years (range)	% with reflux >/= grade 3	Incidence of CKD (upper limit of GFR for CKD in mL/s per 1.73 m^2^)	Incidence of ESRD	Predictors of ESRD/CKD
Ardissino, J Urol, 2004 [21]	322 (245)	>5	95%	N/A—CKD was an inclusion requirement	56%	Proteinuria, CrCl <0.67 mL/s/1.73 m^2^
Caione, BJU Int, 2004 [23]	50 (42)	6.3 (1–16)	100%	54% (1.3)	0%	Creatinine r >53 umol/L in the first year
Neild, BMC Neph, 2004 [24]	44 (22)	NR	Not reported (NR)	N/A—CKD was an inclusion requirement	N/A	Proteinuria, GFR < CrCl <0.83 mL/s/1.73 m^2^
Lahdes-Vasama, NDT, 2006 [25]	267 (58)	37 (27–48)	NR	67% (1.5)	9%	Bilateral scarring
Mor, BJU Int, 2003 [26]	100 (21)	20–30	NR	1% (1.5)	0	NR
Silva, Ped Neph, 2006 [27]	735 (208)	6.3 (0.5–34)	60% of renal units	3.1% (<1.25)	1.5%	Hypertension
Silva, Ped Neph, 2006 [28]	184 (69)	6.5 (1.1–34)	100%	15%	5.4%	Bilateral VUR, grade V VUR, diagnosis before 1990, diagnosis at age >24 months
